# Interactive Effects of an Herbivore-Induced Plant Volatile and Color on an Insect Community in Cranberry

**DOI:** 10.3390/insects11080524

**Published:** 2020-08-12

**Authors:** Cesar Rodriguez-Saona, Pablo Urbaneja-Bernat, Jordano Salamanca, Vanessa Garzón-Tovar

**Affiliations:** 1 P.E. Marucci Center for Blueberry & Cranberry Research, Rutgers University, Chatsworth, NJ 08019, USA; paurbaneja@gmail.com; 2 Escuela de Ciencias Agrícolas, Pecuarias y del Medio Ambiente (ECAPMA), Universidad Nacional Abierta y a Distancia (UNAD), Bogotá 110111, Colombia; jordano.salamanca@unad.edu.co (J.S.); vanessagarzn@gmail.com (V.G.-T.)

**Keywords:** methyl salicylate, PredaLure, natural enemies, *Toxomerus marginatus*, monitoring, biological control

## Abstract

**Simple Summary:**

Plants often increase their odor emissions after herbivore feeding damage, which in turn attract natural enemies of the herbivores such as insect predators. Synthetic versions of these so-called herbivore-induced plant volatiles (HIPVs) can be used to monitor populations of beneficial insects in agriculture. In addition, HIPVs can potentially attract the herbivores themselves. However, whether synthetic HIPVs interact with color to affect insect communities in farms is unknown. In this study, we tested a lure containing the HIPV methyl salicylate (named ‘PredaLure’) in combination with five different colored sticky traps to monitor insect populations in cranberry fields (also known as bogs). We found that hoverflies (also called flower flies or syrphid flies), whose larvae are predators of several insect pests including aphids and thrips, were attracted to PredaLure but this attraction was affected by the color of the trap. In fact, the numbers of hoverflies were 2–4 higher on yellow and white traps baited with PredaLure than on unbaited traps. Irrespective of trap color, plant-feeding thrips were also more attracted to PredaLure-baited than unbaited traps. Our study provides guidelines for the use of odor-baited colored sticky traps to monitor natural enemies such as hoverflies in an agricultural system like cranberries.

**Abstract:**

Synthetic herbivore-induced plant volatiles (HIPVs) could be used to monitor insect populations in agroecosystems, including beneficial insects such as natural enemies of herbivores. However, it is unknown whether insect responses to HIPVs are influenced by visual cues, e.g., color. We hypothesized that the HIPV methyl salicylate (MeSA) interacts with color to affect insect captures on sticky traps. To test this, we conducted a 5 × 2 factorial field experiment in a commercial cranberry farm to monitor numbers of insect predators, parasitoids, and herbivores by using five colored sticky traps that were either baited with a MeSA lure (named ‘PredaLure’) or unbaited. At the community level, PredaLure increased captures of predators. At the individual-taxon level, captures of the hoverfly *Toxomerus marginatus* (Diptera: Syrphidae) and thrips (Thysanoptera: Thripidae) were higher on PredaLure-baited traps. However, only captures of *T. marginatus* on PredaLure-baited traps interacted significantly with color such that the numbers of this hoverfly on yellow and white traps were 2–4 times higher when baited with PredaLure. This study is the first to document the interactive effects of synthetic HIPVs and color on an insect community. Our findings have implications for optimal selection of HIPV-baited colored traps to monitor natural enemy populations in agroecosystems.

## 1. Introduction

Volatile organic compounds (VOCs) emitted from plants play multiple roles in nature. They can protect plants from biotic stresses by priming plant defenses [1,2] or from abiotic stresses such as ozone, temperature, and light [3,4,5,6]. These VOCs are also important in plant–plant interactions [7,8,9] and mutualistic plant–insect interactions by attracting beneficial insects, including pollinators [10], natural enemies of herbivores [11], and seed dispersers [12,13]. However, they can also play a role in antagonistic interactions by attracting insect herbivores [11,14]. VOC emission generally increases when plants are attacked by insect herbivores (so-called herbivore-induced plant volatiles (HIPVs)) [15,16]. For natural enemies, HIPVs are more detectable and reliable cues during herbivore prey/host location than constitutive VOCs [17]. For instance, several insect predators and parasitoids are attracted to HIPVs [16], and this attraction can increase their predation and parasitism rates [18,19,20]. HIPV emissions can also affect plant-pollinator interactions [21] as well as plant-herbivore interactions [19]. Therefore, the emission of HIPVs can have profound effects on insect communities.

Due to the importance of HIPVs in tri-trophic (plant–herbivore–natural enemy) interactions, synthetic versions of them have been used to attract natural enemies (predators and parasitoids) of herbivores in agroecosystems [22,23]. An HIPV commonly used for this purpose is methyl salicylate (MeSA), which is a volatile compound emitted from leaves and flowers of many plant species [10], and that is induced by herbivory from insects belonging to different feeding guilds, such as cell-content feeders [24], sap-sucking feeders [25,26], and chewers [27]. A meta-analysis showed natural enemies have a general attraction to MeSA in agricultural fields, and this attraction was not different among predator and parasitoid taxa [28]. For example, insect predators, including the big-eyed bug *Geocoris pallens* Stal. (Hemiptera: Geocoridae), the lady beetle *Stethorus punctum picipes* (Casey) (Coleoptera: Coccinellidae), the green lacewing *Chrysopa nigricornis* Burmeister (Neuroptera: Chrysopidae), and hoverflies (Diptera: Syrphidae), are attracted to sticky traps baited with MeSA in hop yards [29,30,31]. In grape vineyards, sticky traps in blocks baited with MeSA captured higher numbers of the predators *C. nigricornis*, *S. punctum picipes*, *Deraeocoris brevis* (Uhler) (Hemiptera: Miridae), and *Orius tristicolor* (White) (Hemiptera: Anthocoridae) [32] and the parasitic wasp *Anagrus* spp. (Hymenoptera: Mymaridae) [33] than traps in unbaited blocks. In similar studies, MeSA-baited traps caught higher numbers of the spider *Erigonidium graminicolum* Sundevall (Araneae: Linyphiidae) and the anthocorid *Orius similis* Zheng in cotton [34], and the seven-spotted lady beetle *Coccinella septempunctata* L. in soybean [25], than unbaited traps. A MeSA-containing lure (PredaLure; AgBio Inc., Westminster, CO, USA) is commercially available to growers for the attraction of natural enemies of agricultural pests. PredaLure-baited sticky traps were more attractive to lacewings and *O. tristicolor* than unbaited traps in strawberry fields [35]. In soybean fields, PredaLure increased the attraction of hoverflies and green lacewings to sticky traps and reduced the abundance of soybean aphids [36].

In cranberries (*Vaccinium macrocarpon* Aiton), hoverflies (mainly *Toxomerus marginatus* (Say)), lady beetles, and green lacewings were captured in higher numbers in sticky traps baited with PredaLure than in unbaited traps [28]. In subsequent studies, MeSA, alone or in combination with other HIPVs, increased *T. marginatus* attraction to sticky traps but repelled megaspilid parasitoids [37]. In a 2-year study, PredaLure reliably increased *T. marginatus* captures on sticky traps; however, it also attracted more phytophagous thrips and, in one year, more plant bugs (Miridae) to these traps [38]. Using video recordings in a field setting, we observed lady beetles, *T. marginatus*, and predatory mites near the PredaLures, which resulted in increased predation of sentinel eggs [38].

Previous studies on the behavioral response of natural enemies to HIPVs have used a single trap color, either yellow e.g., [28,29,31,38,39] or white e.g., [35,39]. However, the interactive effects of HIPVs and visual cues, e.g., color, on natural enemy attraction have not been investigated. This knowledge gap is critical considering that visual cues play an essential role in the host-finding process of insects, including predators, parasitoids, and herbivores [17,40]. Thus, we tested the hypothesis that the response of insects to the HIPV MeSA is influenced by color. To achieve this, we conducted a field experiment in cranberries to address the following question: Are captures of insects from three feeding guilds (predators, parasitoids, and herbivores) on MeSA (PredaLure)-baited traps affected by the color of traps? If so, which taxa?

## 2. Materials and Methods

### 2.1. Site and Study Design

**Figure 1:**
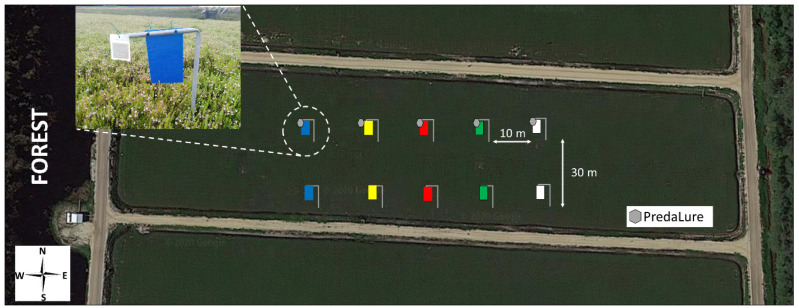
Schematic layout of methyl salicylate (MeSa) (PredaLure)-baited and unbaited color sticky traps in cranberry bogs. Insert photo shows a closeup of a blue PredaLure-baited trap.

A field experiment was performed in 2019 to examine the interactive effects MeSA (PredaLure) and color on insect trap captures in cranberries in southern New Jersey (USA). In New Jersey, cranberries are grown in wet, marshy fields characterized by acidic, sandy soils called bogs (other names include beds or marshes). The experiment was conducted in five (3–4 acre) cranberry, *V. macrocarpon* (cv. ‘Early Black’), bogs in a commercial farm located in the Pinelands National Reserve, Chatsworth, New Jersey (Latitude: 39°44′12.17″ N; Longitude: 74°32′20.71″ W). All bogs were adjacent to a wooded habitat dominated by Pitch pine, *Pinus rigida* Mill., where wild *Vaccinium* plants are typically found in the understory [41] (Figure 1), and each bog was considered a replicate.

In each bog, two sets of five color traps were randomly placed in a straight line and were at least 10 m from the bog’s edge. To avoid positional bias in relation to proximity to the forest habitat (a potential source of natural enemies), traps of the same color were paired so that traps within bogs had the same sequence of colors (Figure 1). The distance between traps within each set was 10 m, and the two sets were at least 30 m apart. One set of 5 color traps was baited with MeSA lures (5 g load/lure; 90 d lure; average release rate of 35 mg/day over a 4-week period at 30 °C constant in the lab; PredaLure, AgBio Inc., Westminster, CO, USA) (insert photo, Figure 1), and the other set of 5 traps had no lures (unbaited controls). Although the position of the colored traps was randomly assigned and changed weekly so that no trap of a particular color was placed in the same position twice during the experiment, color traps always remained paired with each other. The position of the lure and non-lure traps in each pair was, however, not alternated between weeks but their position was randomly assigned among replicates. Traps and PredaLures were placed for 4 weeks, from 11 June until 09 July, which corresponds to the entire bloom period and coindices with peak hoverfly (*T. marginatus*) adult activity [28]. We focused our trapping on the period of *T. marginatus* activity because this insect was consistently the most attracted to MeSA in previous studies [28,37,38]. According to the manufacturing company, PredaLures last for at least 4 weeks in the field, so they were not replaced. None of the bogs had insecticides applied prior to or during the study.

### 2.2. Color Traps

Five color traps were tested: yellow (used as our standard [28,38]), white, green, blue, and red. All traps were 22.8 × 14.0 cm made of (0.24-mm-thick) colored acrylic sheets (Laird Plastics, Bristol, PA, USA; catalog no. 103617, 103416, 207486, 102249, and 103220 for yellow, white, green, blue, and red, respectively). Reflectance data for these colored traps are reported in Silva et al. [42]. Traps were coated on both sides with Tangle-Trap Insect Trap coating (The Tanglefoot Co., Grand Rapids, MI, USA) and were hung vertically with twist ties to 40-cm-high metal poles (see insert photo; Figure 1). Poles were buried in fields such that trap bottoms were <10 cm above the canopy.

### 2.3. Data Collection

During each week of sampling, traps were collected and processed in the laboratory where the numbers of insect predators, parasitoids, and herbivores on traps were counted under a stereomicroscope. The most abundant insect predators, parasitoids, and herbivores were identified to family and, when possible, to species levels. The “predator guild” included the following insect predators (common name (Order: Family)): hoverflies (Diptera: Syrphidae), lady beetles (Coleoptera: Coccinellidae), pirate bugs (Hemiptera: Anthocoridae), brown lacewings (Neuroptera: Hemerobiidae), and predatory thrips (Thysanoptera: Aeolothripidae). Spiders (Araneae) were also counted and included in the predator guild in the data analyses (see below). The “parasitoid guild” included the following nine parasitoid families (Order Hymenoptera): Megaspilidae, Aphelinidae, Encyrtidae, Mymaridae, Trichogrammatidae, Ceraphronidae, Figitidae, Braconidae, and Ichneumonidae. The two most commonly found insect herbivores (“herbivore guild”) were thrips (Thysanoptera: Thripidae) and click beetles (Coleoptera: Elateridae).

### 2.4. Data Analyses

We recorded weekly captures of each insect taxon per trap and performed data analyses as a 5 × 2 factorial, with 5 colors and 2 odor treatments (with or without MeSA). Prior to analyses, the data were checked for normality using the Shapiro–Wilk test [43] and for homoscedasticity using the Levene’s test (‘car’ package in R). At the community level, we performed multivariate analysis of variance (MANOVA) to test for the effects of MeSA (PredaLure) and color on the three insect feeding guilds (predators, parasitoids, and herbivores). The full model included treatment (PredaLure-baited versus unbaited traps), color, the interaction between treatment and color, and block (bog). At the individual-taxon level, we performed analysis of variance (ANOVA) mixed effect models (‘nlme’ package) to analyze the effects of PredaLure, color, and the interaction between them (fixed effects) on each insect taxon, with block and date of sampling as random effects. A significant ANOVA was followed by Tukey’s HSD test (α = 0.05; ‘agricolae’ package in R). If needed, data were transformed before ANOVA using ln(x *+* 0.5) to meet assumptions of normality. Untransformed data are presented in figures. All data analyses were performed in R version 3.4.4 [44].

## 3. Results

### 3.1. Effects of MeSA and Color on Insect Community

Captures of predators on sticky traps were influenced by MeSA (PredaLure) and color, but not by the interaction between them (Table 1). In contrast, captures of insect parasitoids and herbivores on sticky traps were influenced by color but not by MeSA or the interaction between them (Table 1).

### 3.2. Effects of MeSA and Color on Insect Predators

**Figure 2:**
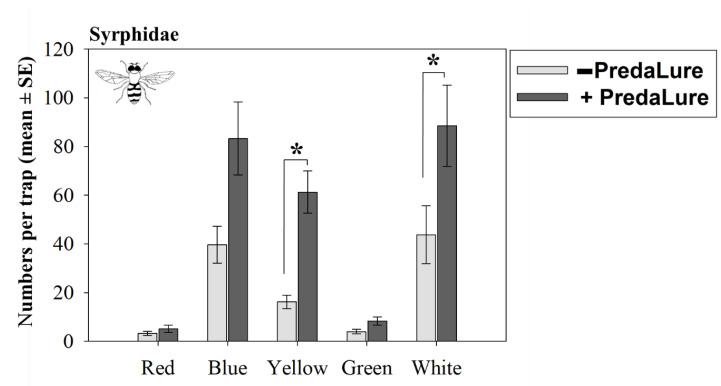
Interactive effects of MeSA (PredaLure) and color on season total numbers (±1 SE) of hoverflies (Syrphidae). An asterisk (*) indicates significant differences between PredaLure-baited and unbaited traps (Tukey’s HSD test, *p* ≤ 0.05).

**Figure 3:**
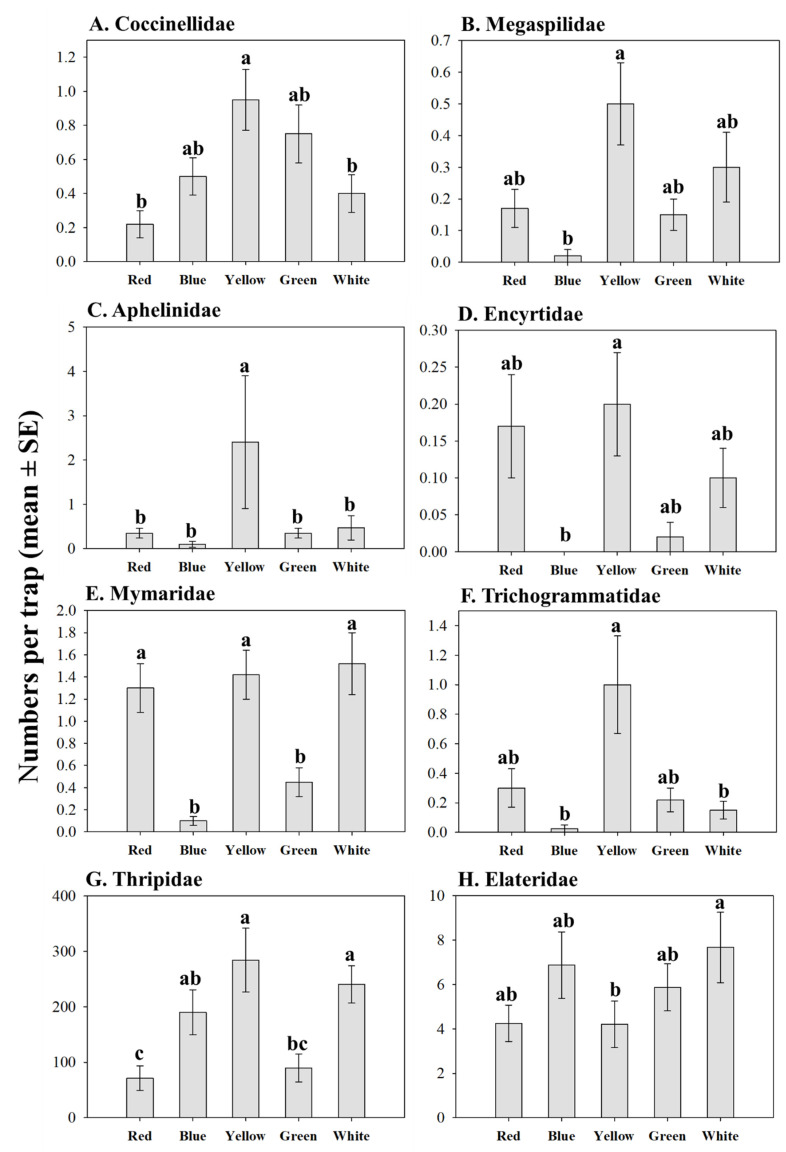
Effects of color on season total numbers (±1 SE) of insect predators (**A**), parasitoids (**B**–**F**), and herbivores (**G**–**H**). Different letters within families indicate significant differences among colors (Tukey’s HSD test, *p* ≤ 0.05).

The color of traps had a significant effect on captures of hoverflies (Syrphidae) and lady beetles (Coccinellidae) (Table 2). The main hoverfly species captured was *T. marginatus*. Blue, yellow, and white traps captured the most *T. marginatus*; whereas green and red traps captured the fewest numbers (Figure 2). Lady beetles were most attracted to yellow and least attracted to red and white (Figure 3A).

Color interacted with MeSA to affect hoverfly captures (significant PredaLure-by-color interaction; Table 2). The numbers of adult *T. marginatus* captured on yellow and white were significantly (2–4 times) higher on PredaLure-baited than on unbaited traps (Figure 2). Although blue traps baited with PredaLure captured 2 times more *T. marginatus* than unbaited blue traps, this difference was only marginal and statistically insignificant (*p* = 0.08; Figure 2). In contrast, the numbers of *T. marginatus* adults captured on red and green traps were similar between PredaLure-baited and unbaited traps.

### 3.3. Effects of MeSA and Color on Insect Parasitoids

Color, but not MeSA or the interaction between MeSA and color, affected the numbers of megaspilid, aphelinid, encyrtid, mymarid, and trichogrammatid wasps (Table 3; Figure 3). In general, these parasitoid families were most attracted to yellow and least attracted to blue (Figure 3B–F).

### 3.4. Effects of MeSA and Color Effects on Insect Herbivores

Color significantly affected captures of phytophagous thrips and click beetles on traps (Table 4; Figure 3). Thrips were most attracted to yellow and white and least attracted to red (Figure 3G), while click beetles were most attracted to white and least attracted to yellow (Figure 3H). Thrips were 1.5 times more attracted to PredaLure-baited than unbaited traps (mean number of thrips per trap [±1 SE]: PredaLure-baited traps = 209.9 ± 24.6; unbaited traps = 144.0 ± 21.3) (Table 4). However, there was no significant interaction between MeSA (PredaLure) and color on thrips captures (Table 4), indicating that these insects responded to PredaLure in a similar manner regardless of color.

## 4. Discussion

For almost four decades, it has been well documented that the natural enemies of herbivores (e.g., predators and parasitoids) use HIPVs during prey/host location [15,16,17,18], which opens the possibility of using synthetic versions of these HIPVs to monitor their populations in agroecosystems e.g., [45,46]. In cranberries (*V. macrocarpon*), this study showed that (1) the HIPV MeSA (PredaLure) increases captures of predators on sticky traps, which was largely driven by an increased attraction of hoverflies (*T. marginatus*) to PredaLure-baited than unbaited traps; (2) *T. marginatus* attraction to PredaLure was influenced by the color of traps (i.e., significant PredaLure-by-color interaction); (3) captures of insect parasitoids on traps were affected by their color but not by the presence of PredaLure; and (4) PredaLure increased the attraction of phytophagous thrips to traps but, unlike *T. marginatus*, this effect was not influenced by trap color (i.e., no significant PredaLure-by-color interaction).

Many insect predators are known to be attracted to MeSA [28]. As in previous studies [28,37,38], we showed a consistent attraction of hoverflies to MeSA (PredaLure) throughout the cranberry flowering period; the most abundant hoverfly species in all these studies was *T. marginatus*. MeSA is known to not only elicit strong antennal responses [47,48], but also increase the attraction of hoverflies in other crops, including hops [29,31], soybean [36], and pear orchards [49], suggesting that physiological (i.e., antennal detection) and behavioral (i.e., attraction) responses of this predatory family to MeSA are common in agroecosystems. MeSA is generally induced by piercing-sucking herbivores, such as aphids [25,26], which are a preferred prey for hoverfly larvae [50,51]. Therefore, it is likely that female hoverflies use MeSA as a cue to locate oviposition sites that maximize immature fitness. To obtain energy while seeking for mates and oviposition sites, hoverflies frequently visit flowers to feed on nectar and pollen [52,53]. Consequently, in addition to biological control services, hoverflies can provide pollination services when visiting cranberry flowers [54]. To locate flowers, hoverflies are thought to primarily use vision, and among visual floral cues, color plays a critical role in this process e.g., [55,56]. In particular, the colors white, yellow, and blue are highly attractive to hoverflies [55,57,58,59]. In agreement with a previous study in cranberries [60], our results showed that *T. marginatus* is most attracted to blue and white, followed by yellow, and is least attracted to red and green. Using similar traps as those used in our study, Silva et al. [42] found that blue and yellow traps have distinct reflectance peaks (blue at 400–500 nm and yellow at 530–700 nm); however, the reflectance peaks for blue and white (at >420 nm) overlapped. Importantly, we found that the response of *T. marginatus* to visual cues (e.g., color) is enhanced by the presence of chemical cues (e.g., MeSA); although, this effect was dependent on the type of color. For instance, *T. marginatus* was 2× more attracted to blue and white and 4× more attracted to yellow in the presence of MeSA. In contrast, the response of this hoverfly to the two least preferred colors, green and red, was not enhanced by MeSA. These results suggest that both visual and chemical cues are likely important in the location and selection process of food and oviposition sites by *T. marginatus*. To our knowledge, this is the first study to document the interactive effects of an HIPV and color on insect predator attraction. Previous studies have shown that combining MeSA with other HIPVs could enhance hoverfly attraction [45,61]; but see [37]. However, whether more complex HIPV blends enhance the response of *T. marginatus* to color needs investigation.

Previous studies in other crops showed attraction of insect parasitoids to MeSA e.g., [28,33]. In our study, however, none of the common parasitoid families (Hymenoptera) found in cranberries were attracted to MeSA. Unlike predators, it is possible that parasitoids need more complex HIPV blends to find their host [62]. De Lange et al. [37] tested the field attraction of natural enemies to MeSA alone and in combination with other HIPVs emitted by cranberries after herbivore feeding damage but found that adding these HIPVs does not change the lack of attraction of parasitoids to MeSA. In contrast, treating cranberries exogenously with methyl jasmonate, a phytohormone involved in HIPV emissions, did increase parasitoid attraction in the field [63]. Altogether, these findings indicate that, in cranberries, the use of HIPVs to manipulate insect parasitoid behavior is a challenging task. In cranberries, all recorded parasitoid families were highly attracted to yellow. Greater numbers of Hymenoptera parasitoids were also caught on yellow sticky traps than on any other colored trap in a previous study in cranberries [60], supporting the importance of visual cues for this beneficial insect group. In recent studies, combining HIPVs with companion plants in a so-called ‘attract-and-reward’ scenario increased the attraction of parasitic wasps [64]. Therefore, finding the ‘right’ attractive HIPV blend that could be used in combination with rewarding (yellow) flowering plants might be needed to enhance and conserve parasitoid populations in habitats near cranberries.

Although HIPV lures like PredaLure are meant to attract natural enemies, insect herbivores can also respond to these lures. In fact, we found that phytophagous thrips were attracted to MeSA-baited traps in cranberries. According to the literature, the behavioral response of phytophagous thrips to MeSA is mixed, possibly due to species identity and crop type [65]. For example, there are reports that MeSA repels and deters oviposition by western flower thrips, *Frankliniella occidentalis* (Pergande) [66,67]. In grapes, however, MeSA had no effects on the abundance of *F. occidentalis* [32]. In another study, grapevines treated with MeSA increased the abundance of phytophagous thrips [68]. In cranberries, higher phytophagous thrips numbers were caught on yellow sticky traps baited with PredaLure than unbaited traps [38]. Phytophagous thrips are also influenced by color [69]. For instance, in cranberries, higher numbers of phytophagous thrips were caught on yellow and white sticky traps than in blue, green, and red traps [60]. Two thrips species, *Frankliniella fusca* (Hinds) and *Scirtothrips* sp., are common in cranberry bogs in New Jersey (C.R-S., pers. observation), and *F. fusca* are most attracted to yellow and white [69], which agrees with results from our current study. At present, thrips are not considered pests of cranberries but are thought to potentially transmit pathogens, such as the tobacco streak virus [70]. However, unlike the predator *T. marginatus*, the attraction of phytophagous thrips to MeSA was not influenced by color.

## 5. Conclusions

In this study, we showed that white and yellow sticky traps baited with the HIPV MeSA (e.g., PredaLure or other similar formulated lures) could be used to monitor populations of the hoverfly *T. marginatus* and thrips in cranberry farms. Yellow and white sticky traps are commercially available and already widely used to monitor insect pest populations in many agroecosystems. Hoverflies provide two important ecosystem services to crops: biological control and pollination. Therefore, by monitoring seasonal fluctuations in hoverfly population size, cranberry growers could increase these ecosystem services through augmentative and conservation biological control. Pest management practices, such as the use of low-risk insecticides and timing and rate of insecticide applications, could be then tailored according to natural enemy trap counts to enhance biological control. To implement MeSA-baited colored traps to monitor hoverfly and thrips populations in cranberries, further studies are needed to estimate their range of attraction.

## Figures and Tables

**Table 1:** Results of multivariate analysis of variance (MANOVA) for the effects of MeSA (PredaLure), color (red, blue, yellow, green, white), and their interaction on predators, parasitoids, and herbivores in commercial cranberry bogs.

Feeding Guild	Variable	Wilk’s λ	df ^a^	*F*	*p* ^b^
Predators	PredaLure	0.89	1, 186	3.53	**<0.01**
	Color	0.53	4, 186	5.23	**<0.001**
	PredaLure × Color	0.85	4, 186	1.18	0.24
	Block	0.83	4, 186	1.36	0.11
Parasitoids	PredaLure	0.97	1, 186	0.57	0.81
	Color	0.48	4, 186	3.97	**<0.001**
	PredaLure × Color	0.86	4, 186	0.75	0.85
	Block	0.84	4, 186	0.86	0.69
Herbivores	PredaLure	0.97	1, 186	2.34	0.09
	Color	0.72	4, 186	8.20	**<0.001**
	PredaLure × Color	0.96	4, 186	0.87	0.53
	Block	2.72	4, 186	2.72	**<0.01**

^a^ Numerator, denominator (error). ^b^ Numbers in bold indicate significant differences at α = 0.05.

**Table 2:** Results of analysis of variance (ANOVA) mixed effect models for the effects of MeSA (PredaLure), color (red, blue, yellow, green, white), and their interaction on predators in commercial cranberry bogs.

Family (Common Name)	Variable	df ^a^	*F*	*p* ^b^
Syrphidae (Hoverflies)	PredaLure	1, 171	25.28	**<0.001**
	Color	4, 171	50.51	**<0.001**
	PredaLure × Color	4, 171	3.79	**<0.01**
Coccinellidae (Lady beetles)	PredaLure	1, 171	0.30	0.58
	Color	4, 171	4.90	**<0.01**
	PredaLure × Color	4, 171	1.05	0.37
Anthocoridae (Pirate bugs)	PredaLure	1, 171	2.61	0.10
	Color	4, 171	1.56	0.18
	PredaLure × Color	4, 171	1.14	0.33
Hemerobiidae (Brown lacewings)	PredaLure	1, 171	1.22	0.26
	Color	4, 171	1.03	0.39
	PredaLure × Color	4, 171	0.73	0.56
Aeolothripidae (Predatory thrips)	PredaLure	1, 171	2.02	0.15
	Color	4, 171	2.17	0.07
	PredaLure × Color	4, 171	2.02	0.09
Araneae (Spiders)	PredaLure	1, 171	2.61	0.10
	Color	4, 171	1.52	0.19
	PredaLure × Color	4, 171	0.32	0.86

^a^ Numerator, denominator (error). ^b^ Numbers in bold indicate significant differences at α= 0.05.

**Table 3:** Results of analysis of variance (ANOVA) mixed effects models for the effects of MeSA (PredaLure), color (red, blue, yellow, green, white), and their interaction on parasitoids in commercial cranberry bogs.

Family	Variable	df ^a^	*F*	*p* ^b^
Megaspilidae	PredaLure	1, 171	0.47	0.49
	Color	4, 171	4.25	**<0.01**
	PredaLure × Color	4, 171	1.22	0.30
Aphelinidae	PredaLure	1, 171	0.91	0.34
	Color	4, 171	6.80	**<0.001**
	PredaLure × Color	4, 171	0.99	0.41
Encyrtidae	PredaLure	1, 171	0.23	0.62
	Color	4, 171	2.95	**0.02**
	PredaLure × Color	4, 171	0.78	0.53
Mymaridae	PredaLure	1, 171	1.68	0.19
	Color	4, 171	16.66	**<0.001**
	PredaLure × Color	4, 171	0.69	0.59
Trichogrammatidae	PredaLure	1, 171	0.26	0.61
	Color	4, 171	7.44	**<0.001**
	PredaLure × Color	4, 171	0.81	0.51
Ceraphronidae	PredaLure	1, 171	0.17	0.67
	Color	4, 171	1.51	0.19
	PredaLure × Color	4, 171	0.63	0.63
Figitidae	PredaLure	1, 171	0.50	0.47
	Color	4, 171	1.95	0.10
	PredaLure × Color	4, 171	0.19	0.94
Braconidae	PredaLure	1, 171	0.73	0.39
	Color	4, 171	1.63	0.16
	PredaLure × Color	4, 171	2.36	**0.05**
Ichneumonidae	PredaLure	1, 171	1.13	0.28
	Color	4, 171	2.24	0.06
	PredaLure × Color	4, 171	0.64	0.63

^a^ Numerator, denominator (error). ^b^ Numbers in bold indicate significant differences at α = 0.05.

**Table 4:** Results of analysis of variance (ANOVA) mixed effects models for the effects of MeSA (PredaLure), color (red, blue, yellow, green, white), and their interaction on herbivores in commercial cranberry bogs.

Family	Variable	df ^a^	*F*	*p* ^b^
(Common Name)				
Thripidae (Thrips)	PredaLure	1, 171	10.53	**<0.001**
	Color	4, 171	32.94	**0.001**
	PredaLure × Color	4, 171	1.48	0.20
Elateridae (Click beetles)	PredaLure	1, 171	3.24	0.07
	Color	4, 171	4.48	**0.001**
	PredaLure × Color	4, 171	1.33	0.25

^a^ Numerator, denominator (error). ^b^ Numbers in bold indicate significant differences at α = 0.05.
